# Clinical, Radiographic, and Histologic Evaluation of Regional Odontodysplasia: a Case Report with 5-year Follow-up

**Published:** 2016-06

**Authors:** Fatemeh Jahanimoghadam, Lida Pishbin, Maryam Rad

**Affiliations:** 1 Oral & Dental Research Center, Dept. of Pediatric Dentistry, School of Dentistry, Kerman University of Medical Sciences, Kerman, Iran.# #; 2 Oral & Dental Research Center, Dept. of Oral Medicine , School of Dentistry, Kerman University of Medical Sciences, Kerman, Iran.

**Keywords:** Regional Odontodysplasia, Ghost Teeth, Dental Dysplasia

## Abstract

Regional odontodysplasia is a developmental anomaly that affects the primary and permanent dentitions. This disorder is generally localized in only one arch and its etiology is still unknown. Clinically, the affected teeth have an abnormal morphology and are typically discolored. Radiographically, these teeth show a ghost-like appearance. This paper reported the results of radiographic, histologic and laboratory findings about the case of a 5-year-old girl presenting this rare anomaly. Her familial history was negative for any genetic anomaly, regional odontodysplasia or other dental anomalies. The patient’s general health was good and no congenital or acquired disease was reported. She was kept under follow-up care until she reached the age of 10 years. Panoramic radiograph showed the involvement of permanent teeth on the right maxillary quadrant. The affected edentulous quadrant was rehabilitated with temporary acrylic maxillary partial denture. The presentation of this case would hopefully have valuable information for pediatric dentists to review the clinical and radiographic features of regional odontodysplasia, yet expediting the diagnosis and treatment of patients with this condition.

## Introduction

Regional odontodysplasia (RO) is a rare developmental dental anomaly affecting both the primary and permanent teeth.[1] The prevalence of this condition is not definitely clear since the studies have been mainly based on the case reports.[2-5] Its prevalence is reported to be less than 1/1000000 and only about 140 cases have been reported in the literature up to the time of this study.[2] It is also known by other terms such as odontogenic dysplasia, ghost teeth, unilateral dental malformation, localized arrest tooth development, familial amelodentinal dysplasia, and odontogenesis imperfecta. This condition affects the tooth structures that originate from the ectoderm and mesoderm. Hitchin described this condition for the first time in 1934.[6] However, the first report that dealt only with the radiographic manifestations of the anomaly was presented by McCall and Wald in 1974.[7] The term odontodysplasia was used by Zegarelli *et al.* in 1963.[8] Since only one quadrant of the jaw is affected by this condition; regional odontodysplasia was selected as the most acceptable term to describe the condition.[1] This condition mostly involves one quadrant and affects the maxilla twice as frequently as the mandible,[9] with the left maxillary quadrant being the most frequently affected quadrant.[10] It has a predilection for the central and lateral incisors, with higher prevalence in females compared to males.[1]

The etiology of this dental anomaly is unknown. However, various factors have been proposed as the etiologic agents including local trauma or infection, teratogenic medications, local disturbances of the blood supply, Rh incompatibility, radiotherapy, neurologic traumas, fever, metabolic and nutritional disorders, and vitamin deficiencies.[10] Heredity seems to play no role in the etiology of this condition because no familial case of such condition has been reported.[11-13]

Nonetheless, this condition has been reported in association with some medical conditions such as vascular nevus, hemangiomas, epidermal nevus syndrome, orbital coloboma, facial hypoplasia on the affected side, hypophosphatasia, ectodermal dysplasia, and hydrocephalus.[14-21]

The diagnosis of regional odontodysplasia depends on clinical and radiographic findings.[22] Clinically, the teeth affected with regional odontodysplasia have an abnormal shape and an irregular super facial contour, with super facial pits and grooves. The teeth are yellow or yellowish-brown in color and appear to be hypoplastic or hypocalcified. The enamel of such teeth is soft when examined with a dental explorer.[23] These teeth are more susceptible to dental caries, very fragile, and they may fracture by minor traumas.[2]

Tooth eruption is delayed and teeth might not even erupt at all. After eruption of the teeth affected with regional odontodysplasia, the most common clinical manifestations are periapical infections and abscess formation even in the absence of dental caries.[2, 22] Radiologically, the affected teeth illustrate abnormal morphology and hypoplastic crown. Additionally, lack of contrast between the enamel and dentin is usually apparent. The enamel and dentin are very thin, displaying a ghost-like appearance.[2, 24] The enlarged pulp chambers, short roots, open apices, and shell-like crowns are the other pathognomonic radiological characteristics.[2, 14]

This report presents a case of regional odontodysplasia that had affected the right quadrant of maxilla in a 5-year-old girl. 

## Case Report

**Figure 1:**
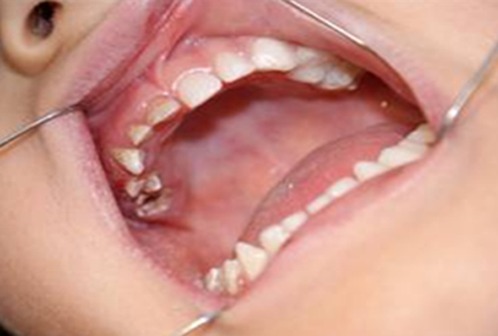
The patient’s intraoral view

A 5-year-old girl was referred to the Department of Pediatric Dentistry, Faculty of Dentistry, University of Medical Sciences, Kerman, Iran with the chief complaint of pain in tooth #55. Her mother had no history of diseases or consumption of any medications during her pregnancy. The child had been born by caesarean section and was the fourth and last child of the family. The mother and father of the child were, respectively, 34 and 41 years old at pregnancy. There was no history of genetic or dental disorders or anomalies in the familial history. The parents were cousins on the maternal side. The child exhibited normal height with below normal weight curve. The patient had normal skin, hair, and general health, with no history of hereditary or acquired medical conditions. Extraoral examinations did not reveal any asymmetry or facial edema. The results of complete blood count (CBC), Alkaline Phosphatase, Ca, P, ferritin and fasting blood sugar (FBS) tests were within normal range. Intraoral examinations did not reveal any oral lesions. Examination of the teeth revealed severe destruction of the deciduous teeth on the right maxillary quadrant (Figure 1) and the teeth #51 and #52 had fistulas.

**Figure 2:**
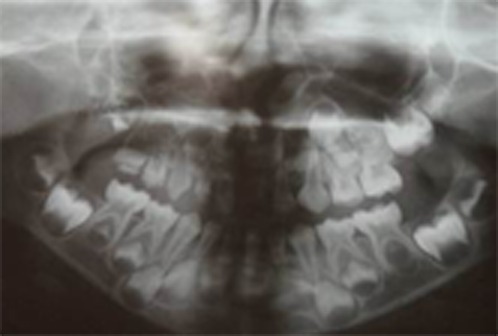
Panoramic radiograph showing the teeth with ghost-like appearance in right maxillary quadrant

Periapical and panoramic radiographs were prepared. In all the primary teeth on the maxillary right quadrant, the pulp chambers and root canals were so large and a very thin layer of hard tissue was seen around the root canals and pulp chambers. The permanent teeth in the same quadrant exhibited the same configuration (Figure 2).

**Figure 3:**
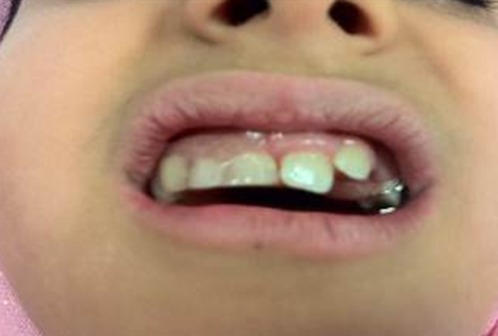
Rehabilitation of the patient with temporary maxillary partial acrylic denture

Based on clinical and radiographic findings, the problem was diagnosed as regional odontodysplasia. With both patient’s and her parents’ consent, the primary teeth on the maxillary right quadrant were extracted due to the pain and infection during a period of 6 months. A temporary acrylic resin prosthetic appliance with a bite plane was fabricated to preserve the alveolar ridge during the period of skeletal growth (Figure 3).

**Figure 4:**
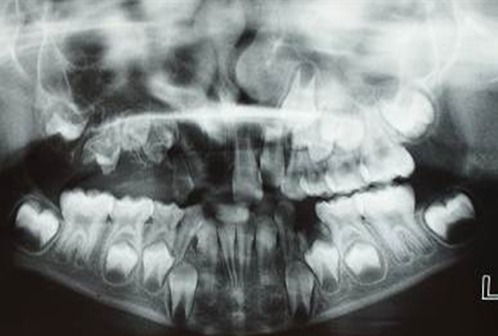
Panoramic radiograph taken during the periodic examinations

Periodic clinical examinations were scheduled due to the possibility of involvement of the permanent teeth on the affected side, as well as for monitoring the eruption of permanent teeth and development of the maxillary arch. Oral hygiene instructions and dietary counseling were performed. The patient was kept under follow-up visits until she was 10 years old. The panoramic radiograph, taken during the periodic examinations, showed the involvement of permanent teeth on the right maxillary side (Figure 4).

**Figure 5:**
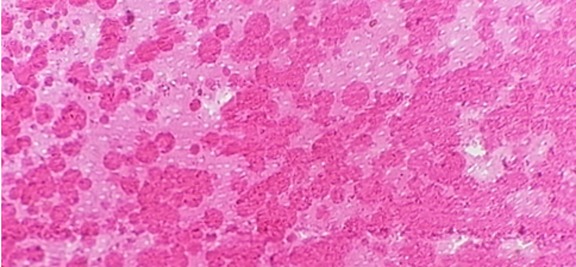
The dentin was atubular in most of the areas with many areas of amorphous material (H & E 400x).

Having obtained the patient’s and parents’ consent, the maxillary right lateral incisor was extracted due to severe pain and fistula. The surgically-removed tooth was histologically examined under an optical microscope in the ground and decalcified section. In most areas, dentin was atubular with many areas of amorphous material (Figure 5). Based on the histopathological features, the provisional diagnosis of regional odontodysplasia was confirmed.

## Discussion

Regional odontodysplasia is a rare developmental anomaly.[1-4] Its exact etiology is uncertain, although several factors are involved.[1-3,25] In many cases, RO is probably misdiagnosed as malformed teeth or odontomas.[26] It occurs in both primary and permanent dentitions, with a marked preference for the maxilla.[27] In the present study, similar to the most previous studies, odontodysplasia affected a maxillary quadrant in a female. Although involving the left side of the maxilla is more common, in this report, the right side of the maxilla was affected. Other conditions such as dentinal dysplasia, shell teeth, hypophosphatasia, dentinogenesis imperfecta, or amelogenesis imperfecta can mimic some features of this anomaly. However, these abnormalities tend to affect the entire dentition.[28] Studies indicated that vascular disorders in RO pathogenicity are very important.[1-4,29] Walton *et al.* reported three cases of RO with vascular nevi overlying the adjacent skin of face.[19] Steiman *et al.* reported a case of RO with vascular nevus in face and neck region.[30] However, it is possible that ischemia which is produced by deficient blood circulation may be a cause.[31] Other causes could be trauma, infection, irritation, vitamin deficiency, genetic, congenital factors, and systemic diseases; moreover, the lesion might be detected in association with hemangioma.[2-4,32-34] In this case report, the patient’s familial history showed neither any congenital or acquired diseases nor was any known factor found. The only findings in this case were the patient’s below-normal weight and her parental consanguinity, which may be related to her disorder. Several factors including the patient’s age, disorder severity, characteristics of the non-involving regions, individual’s aesthetic and function may be considered in odontodysplasia treatment.[35] The patient’s age and cooperation, prognosis of teeth, and follow-up examinations significantly influence the selective treatment. Positive medical history like mental and physical conditions, can affect the child’s cooperation and dental treatment. Besides, these problems would have explicit effects on selecting the treatment plan. Furthermore, the child’s and parents’ attitude and desire towards the treatment plan are very important which influence the decision of performing a conservative treatment with multiple follow-up examinations in a long time or a short-time treatment plan. The number and position of involved teeth are effective in the future problems of prosthetic, restorative, and orthodontic treatment, as well as space management.[2, 25] There is a controversy about the treatment of regional odontodysplasia. These cases need a continuous and multidisciplinary approach. Most clinicians advocate extracting the affected teeth as soon as possible and inserting a prosthetic treatment, whereas other clinicians prefer restorative treatments (if possible) to protect the affected erupted teeth.[27] Selection of a method and its timing appear to be critical factors in the treatment planning of RO. In very young children, the teeth in the arch should be retained, while those involved with abscesses cannot be restored and must be extracted.[36] On the contrary, in older children, the abscessed permanent teeth should be extracted with others must be retained until final rehabilitation with implants or fixed prosthesis.[37] The patient was placed on periodic recall to monitor the growth and development of her dental arches. Unfortunately in the present case, the patient was referred to the Department of Pediatric Dentistry after the involvement of dental pulp, so the prophylactic treatment was not possible. Therefore, pulp therapy was indicated in this situation. Early diagnosis and clinical and radiographic characteristics of these patients are of great importance. If the patient had been referred to the department sooner, prophylactic treatment could have been considered and her teeth would probably have been saved and consequently, the development of alveolar ridge has been expedited.

## Conclusion

Treatment of a child with regional odontodysplasia requires a multidisciplinary approach. Consultations between pediatric, prosthodontic, and orthodontic specialists are necessary to provide the best treatment plan. Treatment planning should be designed separately for each individual case. It depends on many factors such as the patient’s age, medical history, involvement extension, teeth eruption, esthetics, and the development stage of pathology. The presentation of this case adds valuable information for pediatric dentists to review the clinical, radiographic and histological features of RO. Moreover, it would remind them that early referral of these patients would be of great importance for their early treatments. 
